# Development in Esophagectomy for Esophageal Cancer: The Current Standing Point of Robotic Surgery

**DOI:** 10.3390/cancers17111878

**Published:** 2025-06-04

**Authors:** Yosuke Morimoto, Satoru Matsuda, Yuki Hirata, Yuki Hoshi, Masashi Takeuchi, Hirofumi Kawakubo, Yuko Kitagawa

**Affiliations:** 1 Department of Surgery, Saiseikai Yokohamashi Tobu Hospital, Yokohama 230-0012, Japan; m.yosuke0115@gmail.com; 2 Department of Surgery, Keio University School of Medicine, Tokyo 160-8582, Japan; y.hirata19@gmail.com (Y.H.); yuki.hoshi01126@keio.jp (Y.H.); masaty871222@gmail.com (M.T.); hkawakubo@keio.jp (H.K.); kitagawa.a3@keio.jp (Y.K.); 3 Department of Surgery, National Hospital Organization Tokyo Medical Center, Tokyo 152-8902, Japan

**Keywords:** robot-assisted esophagectomy, minimally invasive esophagectomy, esophageal cancer

## Abstract

Despite advancements in multidisciplinary treatment, esophagectomy remains the primary curative treatment for esophageal cancer. Given that lymph node metastases can spread from the cervical to abdominal regions, three-field lymph node dissection has been established as a standard approach. However, this highly invasive procedure involves multiple anatomical regions—thoracic, abdominal, and cervical—leading to significant surgical burden. To reduce surgical invasiveness, minimally invasive esophagectomy (MIE) has become increasingly common worldwide. With its adoption and advancements in multidisciplinary therapy, discussions have emerged regarding the potential omission of lymph node dissection in selected cases. Since the introduction of robot-assisted minimally invasive esophagectomy (RAMIE) in 2004, this technique has progressively replaced conventional MIE. Robotic systems—equipped with a magnified 3D camera, articulated instruments, and tremor filtering—allow surgeons to perform complex procedures with greater precision than manual techniques. One randomized controlled trial (RCT) has demonstrated fewer postoperative complications with RAMIE compared to open esophagectomy. Additionally, RAMIE has been shown to enable more extensive lymph node dissection around the left recurrent laryngeal nerve than conventional MIE. However, the long-term oncological benefits of RAMIE remain unproven, as no RCTs have definitely confirmed its impact on long-term survival in esophageal cancer patients. Ongoing randomized trials are expected to provide further insights into its prognostic benefits.

## Simple Summary

Esophageal cancer is one of the leading causes of cancer-related deaths worldwide. Surgery is the standard treatment for resectable esophageal cancer. However, esophagectomy is a highly invasive procedure that involves the thoracic and abdominal regions, or sometimes, the thoracic, abdominal, and cervical regions. To reduce surgical invasiveness, minimally invasive esophagectomy has become increasingly common worldwide. More recently, robot-assisted minimally invasive esophagectomy has gained popularity. This scoping review provides a comprehensive overview of the development of esophagectomy and the evidence from published and ongoing randomized Phase III trials, shedding light on the current status of minimally invasive esophagectomy, including robot surgery. It has been demonstrated that surgical and perioperative outcomes are superior to those of open esophagectomy or conventional minimally invasive esophagectomy, but the long-term prognosis remains unclear. Ongoing randomized trials are expected to provide further insights into its prognostic benefits.

## 1. Introduction

Esophageal cancer (EC) is highly malignant with a poor prognosis due to its rapid progression, even in early stages [1,2]. Despite advances in multidisciplinary treatments, esophagectomy remains the primary curative approach for EC, including esophageal squamous cell carcinoma (ESCC) and adenocarcinoma (EAC) [3]. Extensive lymph node (LN) dissection is a crucial component of surgical resection, as achieving an R0 resection is essential for long-term survival. Extended lymphadenectomy, or three-field LN dissection, has been developed particularly for ESCC, the predominant type in Asian countries such as Japan, and has been shown to improve survival rates [4]. However, despite advancements in extended LN dissection and perioperative management, esophagectomy remains a highly invasive procedure associated with significant postoperative complications.

To mitigate complications and enhance prognosis, minimally invasive esophagectomy (MIE) has been developed. In recent years, MIE has gained widespread adoption, with robot-assisted surgery becoming increasingly common. According to National Clinical Database data from Japan, the proportion of MIE cases increased from 37.0% in 2012 to 74.8% in 2021 [5]. The trend is also observed in Europe [6]. MIE has evolved from video-assisted thoracoscopic surgery (VATS) to robot-assisted minimally invasive esophagectomy (RAMIE). The da Vinci surgical system (Intuitive Surgical, Sunnyvale, CA, USA) enhances visualization with a magnified three-dimensional (3D) camera and offers full articulation of instruments, providing seven degrees of freedom. Additionally, its tremor filtering and motion scaling enable precise manipulations in confined surgical fields [7]. This review explores the historical evolution of esophagectomy and the development of minimally invasive techniques, including VATS and RAMIE, for treating EC.

## 2. Development of Esophagectomy and LN Dissection for EC

The origins of esophagectomy date back to the 19th century. In 1877, Czerny performed the first cervical EC excision, though without reconstruction [8]. Von Mikulicz achieved the first cervical esophagectomy repair in 1886 [9]. Wookey introduced a two-stage reconstruction using a skin flap following laryngopharyngectomy and cervical esophagectomy in 1942 [10]. In 1959, Seidenberg performed the first free jejunum transfer for cervical EC, a procedure that remains the standard for reconstructing cervical EC today [11].

The first successful thoracic EC surgery was conducted by Torek in 1913 [12]. At that time, reconstruction was not performed, and the patient relied on an artificial rubber esophagus between the esophagostomy and gastrostomy. In 1929, Osawa achieved the first successful single-stage EC reconstruction [13]. In 1946, Ivor-Lewis introduced a two-step surgical approach involving esophagogastric anastomosis in the thoracic cavity after gastric tube formation via laparotomy and right thoracotomy [14]. The Ivor-Lewis procedure remains widely used for lower thoracic EC and esophagogastric junction (EGJ) cancer. In 1954, Nakayama introduced a right thoracotomy with esophagectomy and gastric tube reconstruction via the antethoracic route, which was considered a groundbreaking procedure at the time, with a 5% perioperative mortality rate [15]. By the 1960s, the importance of LN dissection to control locoregional recurrence had gained recognition. In 1963, Logan reviewed mediastinal lymphadenectomy via left thoracotomy, reporting a 15.6% surgical mortality rate [16]. In 1983, Skinner advanced the en bloc esophagectomy with mediastinal LN dissection using a right thoracotomy, which has since become as a standard treatment worldwide [17].

## 3. Oncological Aspects of EC and Lymphadenectomy

The esophagus has an extensive lymphatic network in the submucosal layer, allowing cancer cells to spread rapidly to the LNs. Takeuchi et al. mapped sentinel lymph nodes (SLNs), the first nodes to receive lymphatic drainage from a primary tumor, in patients with superficial ESCC [1]. The authors reported an average of 4.7 SLNs per patient, with locations ranging from the cervical to abdominal regions, regardless of tumor site. Similarly, Akutsu et al. analyzed data from a prospective multi-institutional randomized trial to assess metastatic LN in cT1 ESCC [2]. The authors found that upper mediastinal LN metastases were common in upper thoracic tumors (Ut), whereas lower thoracic tumors (Lt) more frequently spread to abdominal nodes. However, for middle thoracic tumors (Mt), LN metastases were observed from the cervical to the abdominal field. Tachimori et al. further investigated 356 ESCC patients with T1b or T2 disease who underwent a transthoracic esophagectomy with three-field dissection (3FD) [18]. Their findings confirmed a high incidence of upper mediastinal LN metastasis in Mt and Lt cases.

These studies established 3FD, cervico-thoraco-abdominal LN dissection, as a key surgical approach for managing trans-lymphatic metastasis in EC. In the cervical region, the supraclavicular lymph nodes and paracervical esophageal nodes are dissected. During thoracic lymphadenectomy, routine dissection includes lymph nodes around the bilateral recurrent laryngeal nerve, paraesophageal nodes, paratracheal nodes, posterior mediastinal nodes, and supradiaphragmatic nodes. In the abdominal field, paracardial lymph nodes, lymph nodes along the lesser curvature, lymph nodes along the trunk of the left gastric artery, lymph nodes around the abdominal esophagus, and infradiaphragmatic lymph nodes are dissected. Developed in Japan in the 1980s, 3FD has since been recognized worldwide as a standard treatment. Japanese guidelines continue to recommend transthoracic esophagectomy with 3FD for EC [19,20].

A critical component of lymphadenectomy in EC is thoracic duct (TD) resection. While metastatic LN involvement around the TD has been documented, its oncologic significance remains unclear. Udagawa et al. examined 778 patients undergoing transthoracic esophagectomy with TD resection, reporting TDLN metastasis rates of 2.2% in pT1b/pT2 and 10.0% in pT3/pT4 tumors [21]. A follow-up study with a larger cohort conducted by the same institution reinforced these findings [22]. In Europe, where adenocarcinoma is more prevalent, Defize et al. conducted a multi-institutional observational study showing TDLNs in approximately 50% of cases, with a 15% metastatic rate [23]. Our previous research was the first to analyze TDLN metastasis prognosis based on tumor location. While TDLN metastasis incidence was relatively low, prognosis was extremely poor [24]. Notably, none of the patients had solitary TDLN metastases without concurrent LN involvement elsewhere, indicating that TDLNs might not serve as direct sentinel nodes in ESCC. Survival analyses revealed that 75% (9 out of 12) of patients with TDLN metastasis experienced postoperative recurrence.

TD resection in transthoracic esophagectomy allows for simultaneous removal of periesophageal adipose tissue, potentially increasing surgical radicality. Furthermore, as previously reported, TD resection enhances LN yield, including nodes around the recurrent laryngeal nerves [25]. These findings align with our earlier research, indicating that extensive LN dissection with TD resection improves survival, particularly in Stage I ESCC, where surgical resection remains the standard of care [26]. However, the survival benefit of TD resection remains debated. Oshikiri et al. reported no significant differences in 5-year overall or cause-specific survival between TD-resected and TD-preserved groups [27].

To establish standardized criteria for lymphadenectomy, the TIGER study, a global prospective observational cohort involving 50 centers, is currently underway [28]. The study aims to map LN metastases in both ESCC and EAC, particularly in the latter, which has not been extensively studied in a multicenter prospective setting [29,30]. The results will provide critical insights into LN metastasis patterns, histology-specific tumor spread, preoperative diagnostics, neoadjuvant therapy, and survival outcomes.

Radical lymphadenectomy is essential for curative treatment, but it also increases the risk of complications, including aspiration pneumonia [31,32]. Thoracoscopic surgery has enabled paraesophageal LN dissection in the neck during thoracic procedures, reducing surgical morbidity. Given the advancements in perioperative treatment, discussions have emerged regarding whether prophylactic supraclavicular node dissection is necessary. In Japan, the JCOG2013 multicenter randomized controlled study is evaluating esophagectomy with or without prophylactic supraclavicular node dissection [33]. The incidence of supraclavicular lymph node metastasis in the upper and middle thoracic ESCC is relatively low, and some patients can still be salvaged postoperatively if metastases occur. Thus, omitting the prophylactic supraclavicular node may help reduce postoperative aspiration and pneumonia without compromising oncological outcomes.

## 4. Minimally Invasive Surgery for EC

A esophagectomy is a highly invasive procedure associated with a high rate of complications [34]. Patients who experience postoperative complications tend to have poor prognoses. Therefore, minimizing surgical invasiveness remains an important clinical goal.

To minimize surgical invasiveness, Cuschieri performed the first thoracoscopic MIE in 1992, a technique that has since gained widespread acceptance [35]. The smaller incisions reduce postoperative pain, while the magnified thoracoscopic view allows for meticulous LN dissection. However, the complexity of the procedure may prolong operative time. In 1996, Akaishi further advanced the technique by performing a thoracoscopic total esophagectomy with superior mediastinal LN dissection [36].

The first randomized controlled trial demonstrating the benefits of thoracoscopic esophagectomy was the TIME trial, published in 2012 [37]. This study identified pulmonary complications within the first two weeks as the primary endpoint, which were significantly lower in the MIE group (12%) compared to the open surgery group (34%). Follow-up analysis revealed comparable oncological outcomes: 3-year overall survival (OS) was 40.4% in the open group vs. 50.5% in the MIE group, while 3-year disease-free survival (DFS) was 35.9% in the open vs. 40.2% in the MI group. These results confirmed that MIE offers a safe alternative to esophagectomy with an acceptable quality of lymphadenectomy [38].

In 2017, Takeuchi conducted one of the largest propensity score-matched comparison studies using a Japanese nationwide database, analyzing 3515 MIE cases versus 3515 open esophagectomy (OE) cases [39]. No significant differences in 30-day mortality rate (0.9% vs. 1.1%) or the operative mortality rate (2.5% vs. 2.8%) were observed between MIE and OE. The proportion of patients requiring more than 48 h postoperative respiratory ventilation was significantly lower in the MIE group than in the OE group. However, the 30-day reoperation rate was significantly higher in the MIE group than in the OE group. These results indicate that while MIE reduces respiratory complications, it may be associated with a higher reoperation rate.

The MIRO randomized clinical trial evaluated the impact of laparoscopic surgery on esophagectomy. In 2019, Mariette et al. compared a hybrid procedure (laparoscopic gastric mobilization with open thoracotomy) to a fully open one, reporting a lower incidence of major complications at 30 days and higher 3-year OS in the hybrid group compared to the open-procedure group (67% vs. 55%). Since postoperative complications negatively affect survival after surgery, the study concluded that minimally invasive surgery can improve outcomes by reducing surgical invasiveness [40]. A 5-year follow-up study confirmed comparable OS and DFS between hybrid and OE. No statistically significant differences in recurrence rate or location were found between the groups, and major postoperative overall and pulmonary complications were identified as risk factors associated with decreased OS and DFS [41].

Despite these advantages, the optimal abdominal approach for minimally invasive thoracoscopic esophagectomy remains debated. Takeuchi et al. compared laparoscopic and open laparotomy techniques for MIE using Japanese nationwide databases [42]. The results showed no significant difference in pulmonary complications between laparoscopy (20.8%) and laparotomy (22.0%, *p* = 0.25). Similarly, pulmonary complications were comparable between laparoscopic assisted surgery and hand-assisted laparoscopic surgery.

The JCOG1409 trial, a phase 3 study, was the first to evaluate MIE’s non-inferiority to OE in terms of OS [43]. The 3-year OS was 82.0% in the thoracoscopic esophagectomy (TE) group and 70.9% in the OE group, demonstrating non-inferiority in OS in MIE compared to OE (HR 0.64, *p* = 0.000726). The R0 resection rates were higher in the TE group than in the OE group, and the reoperation rate was lower in the TE group than in the OE group. Respiratory dysfunction at three months post-surgery was significantly lower in the TE group than in the OE group. These findings confirmed that TE is a standard treatment for stage I-III thoracic EC.

The Upper GI Oncology Summit 2023 also discussed MIE for EGC cancer, recommending thoracoscopic (or robotic) esophagectomy over OE when a transthoracic approach is indicated [44].

## 5. Robot-Assisted Minimally Invasive Surgery for EC

**Figure 1:**
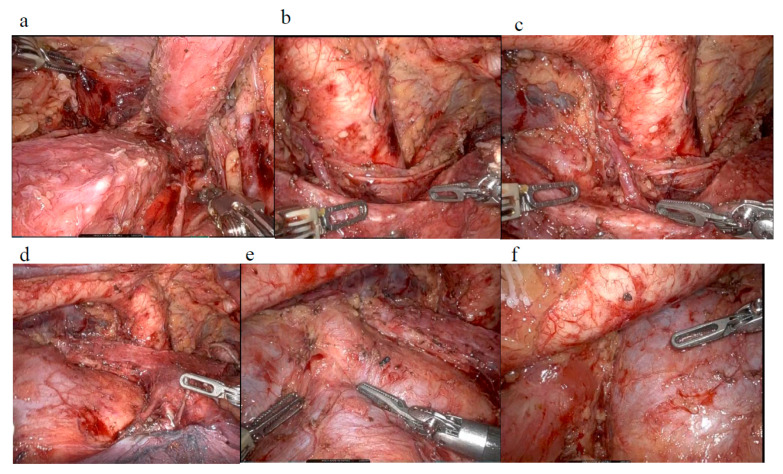
Completion of lymphadenectomy in RAMIE: (**a**) right RLN lymph node dissection; (**b**) left RLN lymph node dissection; (**c**) left tracheobronchial lymph node dissection; (**d**) subcarinal lymph node dissection; (**e**) middle mediastinal lymph node dissection; (**f**) lower mediastinal lymph node dissection.

More recently, RAMIE has gained popularity. In 2004, Bodner performed the first RAMIE using the da Vinci surgical system [45]. The robotic platform offers stabilized robotic forceps with multiple joints for greater precision, and a magnified, high-resolution, 3D view for meticulous dissection. Using robotic assistance during the thoracoscopic phase enhances LN dissection along vital mediastinal structures, thereby improving surgical precision [46]. The diagrams of the completion of lymphadenectomy in RAMIE is shown in Figure 1. A summary of randomized controlled trials on RAMIE is provided in Table 1.

Sluis et al. conducted the ROBOT trial, a phase III randomized study comparing OE and RAMIE in McKeown esophagectomy [47]. A total of 112 patients were randomized, and the key findings revealed lower overall complication rates in the RAMIE group, including significantly fewer cardiac (47% vs. 22%) and pulmonary (32% vs. 58%) complications. Furthermore, RAMIE was associated with reduced blood loss, lower postoperative pain scores, faster functional recovery, and better quality of life. Conversely, similar R0 resection rates, the 5-year OS (41% in the RAMIE group and 40% in the OE group, *p* = 0.827), and the 5-year DFS (42% in the RAMIE group and 43% in the OE group, *p* = 0.749) were noted. Furthermore, the number of retrieved LNs and all pathologic outcomes were comparable between the groups. Although recurrence rates (median to recurrence: 10 months) and patterns were similar between both groups, RAMIE significantly reduced postoperative morbidity at 30 days without compromising long-term oncological outcomes [48]. The ROBOT-2 trial, an ongoing randomized superiority trial, is comparing RAMIE vs. MIE with intrathoracic anastomosis (Ivor-Lewis procedure) in patients with resectable intrathoracic EAC or EGJ adenocarcinoma in Western populations [49]. The primary outcome of this study is the total number of resected abdominal and mediastinal LNs per station.

Chao et al. conducted the REVATE trial, a multicenter randomized clinical trial comparing RAMIE and VATS for left RLN LN dissection [50]. Results showed that the successful left RLN dissection rates were 88.3% in the RAMIE group and 69% in the VATS group (*p* < 0.001). Furthermore, the RAMIE group had a lower incidence of left RLN palsy than the VATS group at one week after surgery, and permanent RLN palsy rates at 6 months were significantly lower in the RAMIE group. Postoperative complication rates were comparable between the two groups, and there were no in-hospital deaths. This trial suggests that RAMIE may improve LN dissection quality and reduce nerve injury compared to VATS.

Yang et al. conducted a randomized controlled trial (RAMIE trial) comparing RAMIE and MIE in ESCC patients [51]. The primary endpoint of this trial is 5-year OS. The short-term outcomes have already been reported. The results showed that RAMIE demonstrated higher left RLN LN dissection success than MIE (79.5% vs. 67.6%, *p* = 0.001) with similar incidences of RLN palsy in patients receiving neoadjuvant therapy. Early results demonstrated that both RAMIE and MIE are safe and feasible for the treatment of ESCC. RAMIE can achieve shorter operative duration and improved LN dissection in patients who received neoadjuvant therapy. The results of the primary endpoint are awaited. Recently, a propensity score-matched study using Japan’s nationwide database compared the surgical outcomes of RAMIE and MIE [52]. The results showed a longer operation time and greater blood loss in RAMIE than in the MIE group. Furthermore, the R0 resection rate was lower in the RAMIE group than in the MIE group, while no differences in the overall complications ≥ Grade IIIa, 30-day mortality rates, and operative mortality rates were noted. Despite longer operative times and higher blood loss, RAMIE and MIE had comparable morbidity rates when performed by skilled board-certified endoscopic surgeons, even in the initial phase of implementation. As robotic techniques improve, RAMIE may outperform MIE in real-world clinical practice.

Several meta-analyses on RAMIE have been published in the past. Meta-analyses comparing RAMIE with OE have shown that RAMIE is associated with significantly lower rates of overall pulmonary complications, pneumonia, atrial fibrillation, and wound infections. It also results in reduced blood loss and shorter hospital stays, although operative times tend to be longer [53]. Furthermore, meta-analyses comparing RAMIE with conventional MIE have demonstrated comparable perioperative outcomes, while suggesting a potential advantage of RAMIE in terms of lymph node dissection in the abdominal cavity, along the left recurrent laryngeal nerve, and in 3-year disease-free survival [54].

**Table 1:** Summary of randomized controlled trials on robot-assisted minimally invasive esophagectomy (RAMIE).

Trial (Ref) (Recruitment)	Country	Approach	Number of Patients	Operation	Type of Carcinoma	Endpoint	Result	*p* Value
ROBOT trial [50]	Netherlands	RAMIE	54	McKnown	AC	Postoperative complications	32 (59%)	0.02
(2012–2016)		OE	55		SCC	(modified Clavien-Dindo classification grade 2–5)	44 (80%)	
RAMIE trial [54]	China	RAMIE	181	McKnown	SCC	Achievement rate of the LND	79.5%	0.001
(2017–2019)		VATS	177			along the left RLN	67.6% (NAC cases)	
						5-yr OS (primary endpoint)	Ongoing	
REVATE trial [53]	Taiwan	RAMIE	51	McKnown	SCC	Success rate of the LND along	88.3%	<0.001
(2018–2022)	China	VATS	51			the left RLN	69%	
ROBOT-2 trial [52]	Germany	RAMIE	109	Ivor-Lewis	AC	Total number of resected LN	Ongoing	-
(2021–ongoing)	Netherlands	VATS	109					
	Switzerland							

RAMIE, robot-assisted minimally invasive esophagectomy; OE, open esophagectomy; VATS, video-assisted minimally invasive esophagectomy; AC, adenocarcinoma; SCC, squamous cell carcinoma; LND, lymph node dissection; RLN, recurrent laryngeal nerve; NAC, neoadjuvant chemotherapy; LNs, lymph nodes; OS, overall survival.

While RAMIE may improve postoperative outcomes, its economic benefits remain unclear. Two propensity score-matched retrospective studies found that although the RAMIE group demonstrated a lower postoperative incidence of recurrent nerve paralysis, the operation times were longer and the hospitalization costs (including surgical costs) were higher compared with MIE [55,56]. Goense et al. examined perioperative medical costs up to 90 days after surgery in the RAMIE and OE groups as a secondary analysis of the ROBOT trial [57]. Although surgical costs were higher in the RAMIE group, overall perioperative medical costs were similar between the two groups (RAMIE: €40,211 vs. OE: €39,495; *p* = 0.932). The authors reported that postoperative complications were the primary driver of increased medical costs across the study population. In studies involving other cancers, robotic surgery has been linked to lower complication rates and improved cost-effectiveness by reducing hospital stays and enhancing recovery [58]. Therefore, if further improvements in clinical outcomes are demonstrated with RAMIE, its high surgical costs may be offset. To date, all studies evaluating the cost-effectiveness of RAMIE have been single-center analyses. Large-scale RCTs are warranted to further assess its economic impact alongside its clinical usefulness.

## 6. Conclusions

In summary, the results of these RCTs suggest that lymphadenectomy around the recurrent laryngeal nerve may be beneficial, with the potential to reduce complications. However, the effect on survival and its economic benefits remains unclear, and further investigation is needed to determine whether this procedure can contribute to long-term prognostic improvement with potential cost-effectiveness.

## 7. Future Perspectives

Advancements in lymph node dissection techniques have improved surgical outcomes in EC. The introduction of RAMIE has further reduced surgical invasiveness, and multiple robotic platforms are becoming available, promising enhanced outcomes. However, training complexities arise with evolving techniques, and thus a standardized robotic training system is necessary to ensure surgical proficiency. Additionally, transcervical approaches aim to minimize surgical invasiveness further. Previously performed via mediastinoscopy, these procedures are now increasingly robot-assisted, enhancing safety and accuracy in esophagectomy [59,60,61]. Furthermore, the transcervical approach using the da Vinci SP system has already been introduced and it is expected that this approach will expand to patients in patients who are not tolerable to the transthoracic approach [62]. With continued advancements in robot-assisted surgery and multidisciplinary treatments, further improvements in EC survival rates are anticipated.

## Data Availability

No new data were created or analyzed in this study. Data sharing is not applicable to this article.
